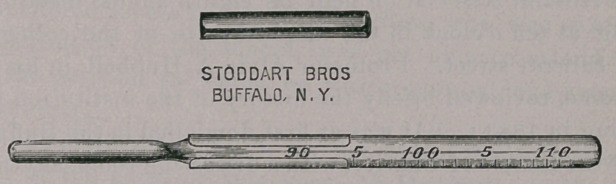# New Instruments

**Published:** 1893-06

**Authors:** 


### New Instruments.

### A SHIELD TO PROTECT THE CLINICAL THERMOMETER.

By Herbert U. Williams, M. D., Buffalo, N. Y.

This device is intended to protect the thermometer while it is being held in the mouth so that it may not be bitten. It consists of a tube of German silver, an inch and a sixteenth (twenty-seven millimeters) in length, having a narrow opening in front. It should clasp the thermometer with moderate pressure, and should slip off easily. It may be made to fit thermometers of various calibers within narrow limits. However, the tubes of thermometers vary so much that it has been found necessary to make the instrument in three sizes. The metal is of such thinness that the shield will go into the case with the thermometer. When not in use it may be put on the end opposite the bulb, where it is out of the way. When it is to be used, the shield should be placed over the lower end of the stem of the thermometer, a quarter to half an inch above the bulb. The working of the thermometer is not interfered with, while the metal prevents the glass beneath from being bitten. It may be removed to be cleaned or boiled.

It may be applied to the lens-front thermometer by broadening the opening in the shield so that the blades embrace the lens. In this case the thermometer should be held in the mouth sideways, in order that the teeth may not bite the lens through the broadened opening.

The shield was designed particularly for taking the temperatures of children. It allows us to do that in the mouth in many cases where we should otherwise have to use the axilla. It is not so useful for very young children as for those old enough to act intelligently, yet whom we are afraid to trust with unprotected thermometers in their mouths.

The shield was made for me by Stoddart Brothers, of Buffalo.

186 Allen Street.

## Figures and Tables

**Figure f1:**